# Influence of Calcareous Deposits on Hydrogen Embrittlement Susceptibility of Q460 Steel

**DOI:** 10.3390/ma17051110

**Published:** 2024-02-28

**Authors:** Xilin Xiong, Haichun Yang, Tongqian Chen, Na Zhang, Tong Niu

**Affiliations:** 1 Institute for Advanced Materials and Technology, University of Science and Technology Beijing, Beijing 100083, China; m202121425@xs.ustb.edu.cn; 2 Qingdao ZITN Micro-Electronics Co., Ltd., Qingdao 266114, China; young17853261300@163.com; 3 School of Materials Science and Engineering, Chongqing University of Technology, Chongqing 400054, China; fengyi@stu.cqut.edu.cn; 4 NCS Testing Technology Co., Ltd., Beijing 100081, China; niutong@ncschina.com

**Keywords:** Q460 steel, calcareous deposits, slow strain test, hydrogen embrittlement susceptibility

## Abstract

Cathodic protection is widely used to protect structural steel from corrosion in marine environments. However, an inappropriate cathodic potential may lead to hydrogen embrittlement (HE). Therefore, this study investigates the relationship between cathodic protection potential, structure and composition of calcareous deposits, and hydrogen embrittlement susceptibility of Q460 steel. The slow strain rate test results and fracture analysis reveal that Q460 steel had the smallest HE susceptibility when covered with the calcareous deposits formed under −1.1 V_SCE_. The deposits have a relatively thin calcium-rich inner layer and a condensed magnesium-rich outer layer, which can significantly inhibit hydrogen entry. A sustained deposition reaction during slow strain rate testing (SSRT) in artificial seawater can also decrease the HE susceptibility of Q460 steel.

## 1. Introduction

With the increasing demand for ocean energy resources, many high-strength low-alloy steels were used in offshore exploration structures. Cathodic protection was used to prevent the steel from corrosion in the marine environment. Under cathodic protection, oxygen reduction (1) and water dissociation (2) reactions occurred on the steel surface, as well as calcareous deposition as in Reactions (3)–(5).

(1) $$O_{2}+{2H}_{2}O+{4e}^{-}\to 4OH^{-}$$

(2) $$H_{2}O+M+e^{-}\to MH_{ads}+OH^{-}$$

(3) $$2OH^{-}+{Mg}^{2+}\to Mg{\left(OH\right)}_{2}↓$$

(4) $$OH^{-}+HCO_{3}^{-}\to H_{2}O+CO_{3}^{2-}$$

(5) $$CO_{3}^{2-}+{Ca}^{2+}\to CaCO_{3}↓$$

A too-negative cathodic protection potential could convert Reaction (1) into Reaction (2) and cause hydrogen adsorption and permeation, and then HE would occur [1,2,3,4,5]. 

The cathodic protection potential is only one factor influencing the sensitivity of steel’s HE in the marine environment. The calcareous deposits referred to as Reactions (3)–(5) also influenced the HE susceptibility, but this situation is complicated. The cathodic protection potential influences the composition of calcareous deposits by changing the metal/electrolyte interfacial pH. Neville et al. denoted that calcite and aragonite formed when the interfacial pH reached 7.4 or greater, and brucite preferred to form when the interfacial pH was greater than 9.5 [6]. Xu et al. found that an interfacial pH of 9.61 at −0.9 V_SCE_ was the critical pH for the formation of magnesium hydroxide, and an inner-brucite layer and an outer-aragonite layer were found to form at −1.0 to −1.2 V_SCE_ [7]. This double-layer structure of calcareous deposits was characterized by a few researchers [2,7,8], but our unpublished work revealed that this double-layer structure is composed of a calcium-rich inner layer and a magnesium-rich outer layer [9].

On the other hand, the composition of calcareous deposits influences hydrogen evolution reaction (HER), and hydrogen permeation, regarded as the first process of HE, was proved to change with the composition of the calcareous deposits. For HER, Gao’s research showed that the hydrogen evolution potential increased with the increase in pre-deposition cathodic potential in the range of −1.05 to −0.90 V_SCE_, which means that the calcareous deposits formed at −1.05 V_SCE_ inhibited HER on the 16 Mn steel comparing with that formed at −0.90 V_SCE_ [10]. Meanwhile, Okstad claimed that the calcareous deposits on carbon steel specimens catalyzed HER [11]. 

Only a few works reported the influence of calcareous deposits on structural steel’s HE. In alkaline artificial seawater, Simoni et al. showed that double-layer structured calcareous deposits formed under −1000 mV_SCE_ did not sensitize HE of API 5 CT grade P110 steel, but those that formed under −1500 mV_SCE_ did [2]. Tian et al. claimed that the HE susceptibility of E690 steel had a linear relationship with the logarithm of hydrogen concentration when cathodic protection was applied in artificial seawater [12]. Zucchi et al. found that HE of 2205 duplex steel increased with the cathodic potential moving negatively from −0.9 to −1.0 V_SCE_ in acidic artificial seawater, but it was reduced at −1.2 V_SCE_ caused by the formation of calcareous deposits [13]. The same point of these works is that they use SSRT in artificial seawater with applied cathodic potential to characterize the HE of specimens. Such an experimental method induced a sustained deposition reaction during the SSRT process, constantly changing the structure and composition. Thus, it is hard to determine the influence of the specified structure and composition of calcareous deposits on HE. 

Above all, the combined effect of cathodic protection potential and calcareous deposits on HE remains unclear. This combined effect was investigated in the present work. SSRT of Q460 steel specimens covered with pre-deposited calcareous deposits were performed in air, 3.5% NaCl solution, and artificial seawater. The fracture analysis was conducted with SEM.

## 2. Experimental

### 2.1. Material and Solution

A 20 mm thick hot-roll low carbon bainite steel Q460 steel provided by Baoshan Iron & Steel Co. Ltd., Shanghai, China was used in this research, and its composition was as follows (wt%): 0.13 C, 1.36 Mn, 0.25 Cr, 0.18 Mo, 0.024 V, 0.008 B, 0.01 P, 0.0031 S, 0.002 Ti. The specimens for microstructure observation were mechanically abraded with SiC grinding paper down to 2000 grit. The microstructure of Q460 steel was observed with an optical microscope after etching with 4 vol.% nital solution. Electron back-scattered diffraction (EBSD) specimens were prepared by electropolishing. The characterization of the microstructure of Q460 steel and fracture morphology was using a Zeiss Auriga EBSD in Beiing, China. 

The artificial seawater described as ASTM D1141-98 and 3.5% NaCl solution was used under different experimental conditions [14]. The composition of the artificial seawater is shown in Table 1. The pH of the artificial seawater was adjusted to 8.2 using a 0.1 M NaOH solution. All the chemical reagents were provided by Sinopharm Group, Beiing, China.

### 2.2. Calcareous Deposition Experiments

**Figure 1:**
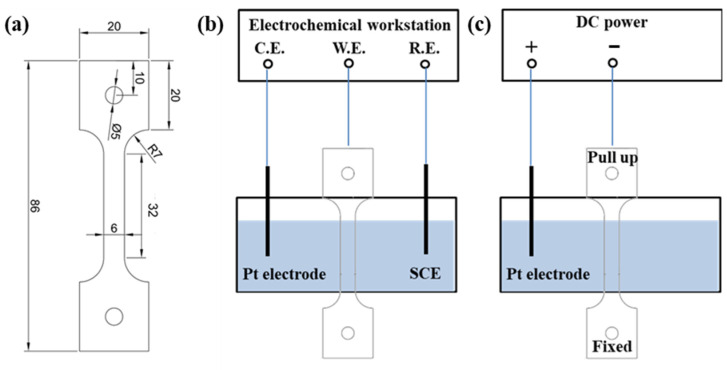
(**a**) Tensile sample geometry in mm (thickness = 2 mm). (**b**,**c**) a Schematic diagram of the equipment of the cathodic deposition experiment and in situ tensile test under ongoing hydrogen charging, respectively. C.E., W.E. and R.E. mean counter electrode, work electrode and reference electrode, respectively.

Cathodic deposition experiments were performed in the artificial seawater with a three-electrode system. A platinum plate and saturated calomel electrode (SCE) were adopted as the counter and reference electrodes. The cathodic deposition experiments were performed before the slow strain rate tensile test with the specimens shown in Figure 1a. The specimens were machined along the rolling direction based on ASTM E8M [15] and mechanically abraded with SiC grinding paper down to 2000 grit, rinsed with distilled water, and cleaned with ethyl alcohol. The standard distance of the specimens was exposed in the artificial seawater. The other parts of the specimens were sealed with silicone. The schematic of the equipment is shown in Figure 1b. The cathodic deposition potentials (−1.0, −1.1, −1.2, and −1.3 V_SCE_) were applied to the specimens by Gamry Interface 1000 (provided by Gamry Instrument, Warminster, PA, USA) electrochemical workstation for 72 h to form calcareous deposits. All of the deposition experiments were performed in artificial seawater at 25 °C.

### 2.3. SSRT

SSRT was conducted with the same specimens as those for cathodic deposition experiments. The schematic of the equipment for SSRT is shown in Figure 1c. Four groups of SSRT experiments were designed in this study, shown in Table 2. Group I was used to obtain the mechanism property of this material in air. There were four specimens in each group of Groups II to IV. Specimens 1 to 4 correspond to performing cathodic deposition experiments from −1.0 to −1.3 V_SCE_. Group III and IV specimens were under hydrogen charging at 0.5 mA cm^−2^ in 3.5% NaCl and artificial seawater.

Comparing Group I and II, the influence of the hydrogen atoms induced by cathodic deposition experiments on the mechanism property of Q460 steel can be determined. Comparing Groups I and III, the effect of the calcareous deposits formed under different cathodic deposition potentials on HE susceptibility can be determined. Comparing Groups III and IV, the effect of persistent deposition during SSRT in artificial seawater on the HE susceptibility of Q460 steel can be determined. Scanning electron microscopy (SEM) was performed to analyze the fracture morphology. The elongation loss rate ($I_{\delta }$) and brittle zone area rate ($I_{S}$) were used to identify the HE susceptibility, shown in Equations (6) and (7), where $\delta_{\mathrm{air}}$ and $\delta_{H}$ are the elongation index of specimen in air and test solutions, respectively, and S and $S_{\mathrm{brittle}}$ are the total area of the fracture of the specimens in solution and $S_{\mathrm{brittle}}$ are the area of the brittle zone in the fracture. The SSRTs were carried out with a nominal strain rate of 1 × 10^−6^ s^−1^ at 25 °C.

(6) $$I_{\delta }=\frac{\left(\delta_{\mathrm{air}}-\delta_{H}\right)}{\delta_{\mathrm{air}}}\times 100\%$$

(7) $$I_{S}=\frac{S_{\mathrm{brittle}}}{S}\times 100\%$$

## 3. Results and Discussion

### 3.1. Microstructure Characterization

**Figure 2:**
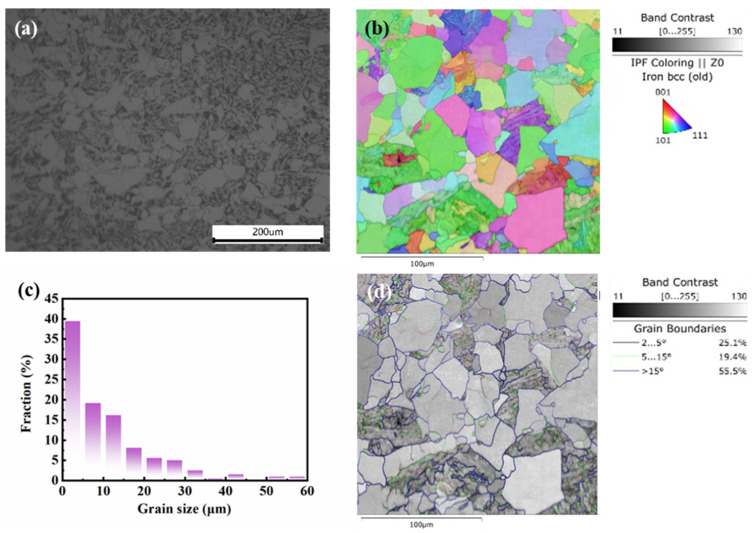
Microstructure characteristics of Q460 steel used in this work: (**a**) optical microscope micrograph of microstructure, (**b**) inverse pole figure of grains, (**c**) statistics of grain size, (**d**) grain boundary map.

The microstructure of Q460 steel used in this work is shown in Figure 1. The optical microscope images in Figure 2a revealed the ferritic-bainitic dual-phase structure. Figure 2b,d give the inverse pole figure and grain boundary features of Q460 steel, respectively. Here, 44.5% of the grain boundaries are low-angle grain boundaries ($\theta <15^{{}^{\circ}}$), and 55.5% of the grain boundaries are high-angle grain boundaries ($\theta >15^{{}^{\circ}}$). The grain size diameter distribution provided by EBSD gives an average grain size value of 11.40 μm. The statistics are shown in Figure 2c.

### 3.2. Influence of Calcareous Deposits on HE Behavior

**Figure 3:**
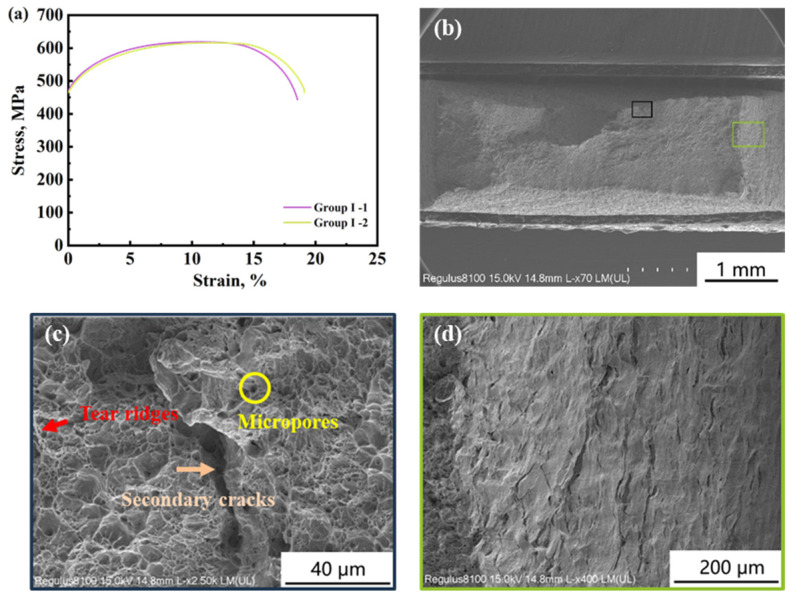
Stress-strain curves of Group I in air (**a**) and the typical fracture morphologies (**b**–**d**).

Two specimens were used in Group I, and SSRT was conducted in air without cathodic deposits. Figure 3a shows the stress–strain curve of the two parallel specimens in Group I. The mechanic properties of the specimens in each group are shown in Table 3. The average yield strength (YS), ultimate tensile strength (UTS), and elongation index (EI) are 467.63 MPa, 616.65 MPa, and 22.30%, respectively. Figure 3b shows the macro morphology of the fracture. Figure 3c,d show the dimple fracture zone and final fracture zone, respectively. A ductile fracture is observed with dimples, micropores, and a few tear ridges, which means a small amount of the quasi-cleavage fracture exists. 

**Figure 4:**
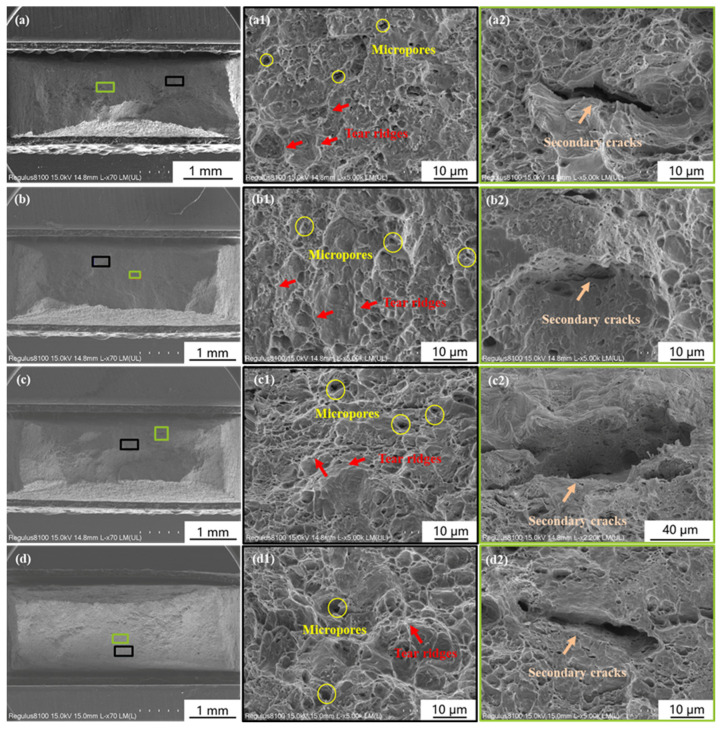
Morphologies of the fracture surface of the specimens in Group II. (**a**–**a2**,**b**–**b2**,**c**–**c2**,**d**–**d2**) corresponding to the specimen 1 to 4 in Group II, which were performed cathodic deposition in the artificial seawater at −1.0 to −1.3 V_SCE_ before SSRT in air, respectively.

Cathodic deposition experiments were performed on the specimens in Group II before SSRT in the air. This group was conducted to measure the influence of hydrogen atoms that permeated during cathodic deposition experiments on the HE susceptibility of Q460 steel. Figure 4 showed the fracture morphologies of the specimens covered with deposits formed under −1.0 to −1.3 V_SCE_, respectively. Similar to the fracture of Group I, a ductile fracture with tear ridges, micropores, and secondary cracks was observed. It can be found that the dimples in Figure 4(d1) are larger than those in Figure 3c, revealing the increase in ductility after cathodic deposition under −1.3 V_SCE_ [16]. According to the stress–strain curves of Group II in Figure 7a and Table 3, it can be observed that the elongation of the specimens of Group II is larger than that of Group I. This phenomenon has been regarded as the hydrogen-enhanced localized plasticity (HELP) in literature [17,18,19,20]. Miresmaeili et al. illustrated that hydrogen atoms promote dislocation emission, motion, and multiplication in hydrogen-rich regions and decrease local flow stress [21]. 

**Figure 5:**
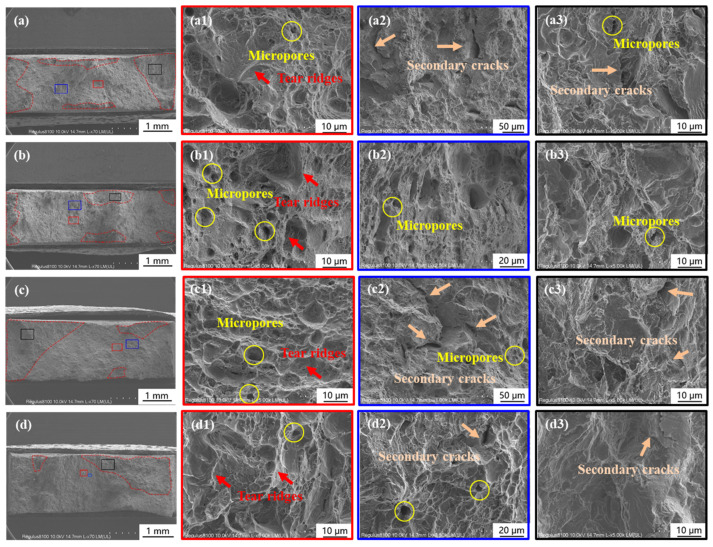
Morphologies of the fracture surface of the specimens in Group III. (**a**–**a3**,**b**–**b3**,**c**–**c3**,**d**–**d3**) corresponding to the specimen 1 to 4 in Group III, which were performed cathodic deposition in the artificial seawater at −1.0 to −1.3 V_SCE_, respectively, before SSRT in 3.5% NaCl solution with 0.5 mA/cm^2^ hydrogen charging current density.

For Group III, calcareous deposits were formed on the specimens’ surface before SSRT in 3.5% NaCl solution with hydrogen charging under 0.5 mA cm^−2^ current density. According to this group, the effect of different kinds of calcareous deposits on HE could be distinguished. Figure 5 shows the fracture morphologies of specimens in Group III. The brittle zone can be observed in the area with red dash lines in Figure 5a–d and the zoom magnified picture in Figure 5(a3–d3). River pattern, secondary cracks, and micropores indicated the occurrence of cleavage fracture. Figure 5(a1–d1) showed the ductile fracture morphologies of the center area of the specimens, and fewer tear ridges were found in Figure 5(b1) than in the other three specimens. Figure 5(a2–d2) showed a quasi-cleavage fracture area, in which the fracture model transfers from ductile to brittle, and the dimples can still be found in Figure 5(b2). Combined with the stress–strain curve of Group III in Figure 7b, a more excellent elongation also means that the specimen Group III-2 covered with the deposits formed under −1.1 V_SCE_ had better ductility. Thus, the deposits formed under −1.1 V_SCE_ could reduce HE susceptibility than others.

**Figure 6:**
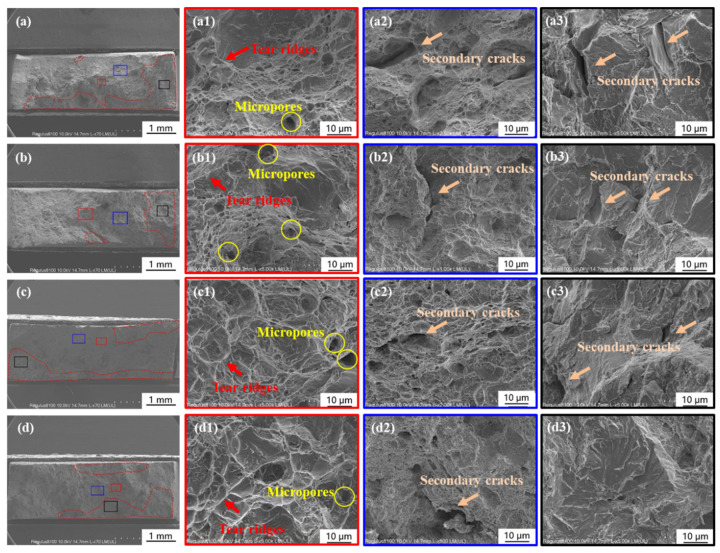
Morphologies of the fracture surface of the specimens in Group IV. (**a**–**a3**,**b**–**b3**,**c**–**c3**,**d**–**d3**) corresponding to specimen 1 to 4 in Group IV, which performed cathodic deposition in the artificial seawater at −1.0 to −1.3 V_SCE_, respectively, before SSRT in artificial seawater with 0.5 mA/cm^2^ hydrogen charging current density.

**Figure 7:**
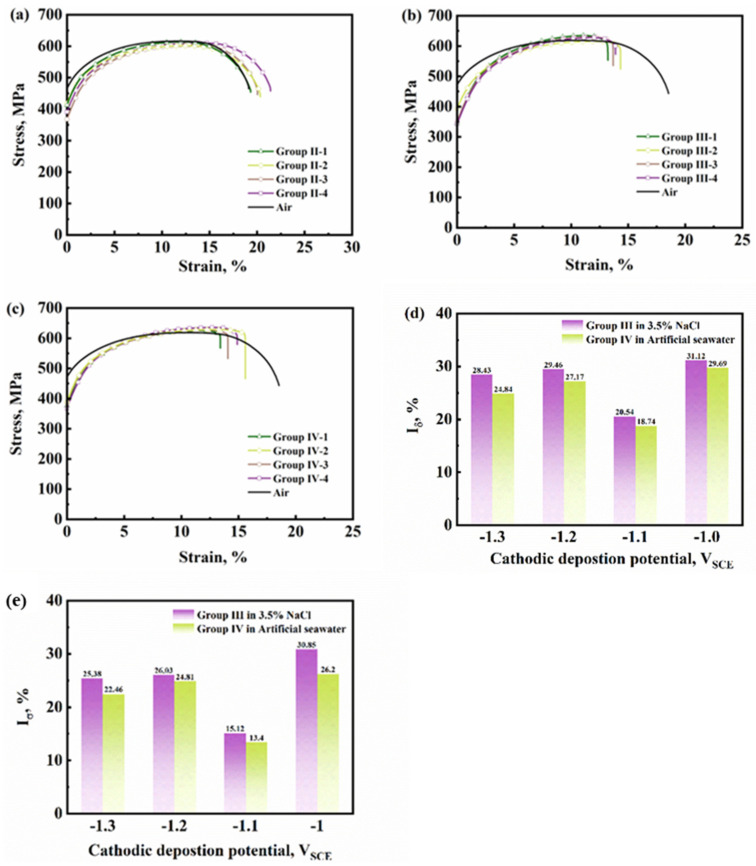
Stress–strain curves of Group II-IV (In each group, the cathodic deposition experiments were conducted with specimens 1 to 4 at −1.0 to −1.3 V_SCE_ in artificial seawater before the SSRT, respectively) (**a**–**c**), and the HE susceptibility indexes in terms of elongation loss (**d**) and brittle zone area rate (**e**) of Q460 steel with hydrogen charging current density of 0.5 mA cm^−2^ in different solution.

Different from Group III, after cathodic deposition experiments, the specimens in Group IV were performed SSRT in artificial seawater with 0.5 mA/cm^2^ hydrogen charging current density. The fracture morphologies are shown in Figure 6. The center area also showed ductile fracture, with micropores, secondary cracks, and tear ridges. The zoom magnified figure in Figure 6(a3–d3) showed cleavage fracture. The elongation loss rate ($I_{\delta }$) and brittle zone area rate ($I_{S}$) of Groups III and IV are shown in Figure 7d,e. A lower ductility loss was observed on the specimens covered with the deposits formed under −1.1 V_SCE_. Comparing Group III with Group IV, both the value of $I_{\delta }$ and $I_{S}$ are smaller when SSRT is conducted in artificial seawater. This difference is caused by the deposits formed during SSRT in artificial seawater. This phenomenon suggests that the deposits inhibit hydrogen entry during SSRT.

An interesting phenomenon was observed in Table 3. For the value of UTS, the specimens in Groups III and IV slightly increased compared with those in Groups I and II. These results are also shown in Figure 12 in Tian and colleagues’ work [12]. By performing SSRT with E690 steel in acid artificial seawater with 10^−2^ mol/L thiosulfate under different cathodic potentials, they also found an increase in UTS, mainly when SSRT was performed under −1050 mV_SCE_. The mechanism of the increase in UTS will be analyzed in future work. On the other hand, the YS of the specimens in Group II-IV are smaller than that in Group I. Some references have mentioned this phenomenon as hydrogen-enhanced macroscopic ductility (HEMP) [22,23,24]. As we mentioned before, the elongation of the specimens of Group II is larger than that of Group I. The difference between HELP and HEMP is that HELP focuses on the interaction of hydrogen atoms and mobility of dislocation, while HEMP focuses on the macroscope ductility. Both of them point out that hydrogen atoms increase ductility in specific circumstances.

**Figure 8:**
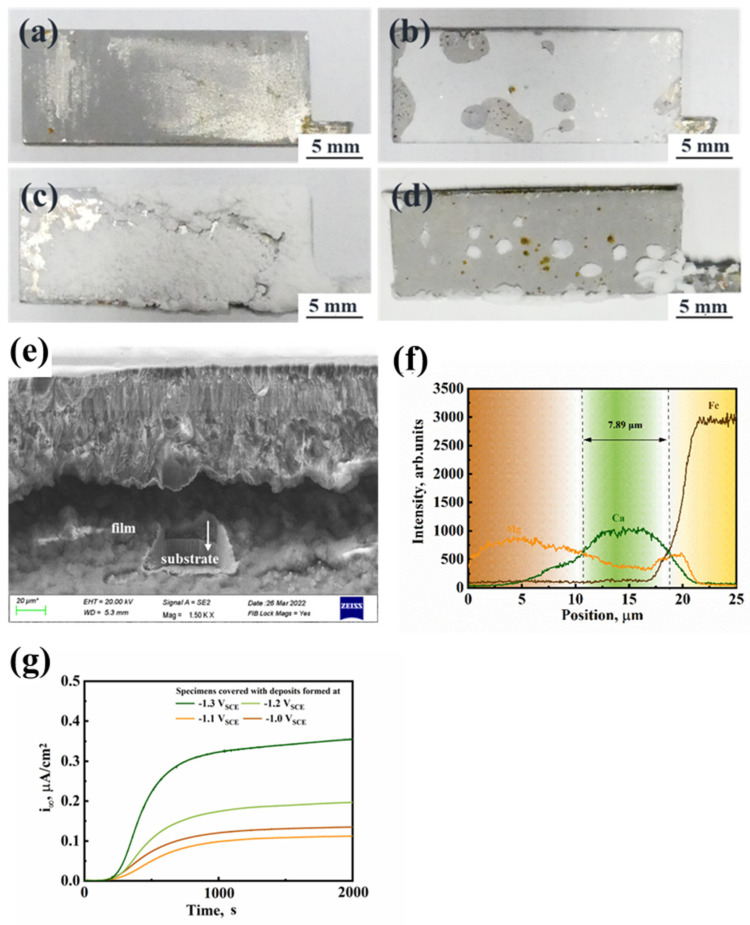
(**a**–**d**) are the macro morphology of the calcareous deposits formed in artificial seawater under −1.0 to −1.3 V_SCE_; (**e**) is the cross-section of the deposits in (**b**); (**f**) is the energy dispersive X-ray spectroscopy of the cross-section of the deposits in (**b**), the faint yellow layer at the right side, the green layer in the middle and dark yellow layer at left side means the matrix, Calcium-rich layer and magnesium-rich layer, respectively; (**g**) is the Devanathan–Stachurski double cell hydrogen permeation tests of specimens covered with deposits on the hydrogen entry side in 0.2 mol/L NaOH solution, and a constant cathodic overpotential of −840 mV was applied on the hydrogen entry side [9].

Our unpublished work systematically characterized the composition and structure of the deposits [9]. The cathodic deposition experimental condition is the same as in Section 2.2. The macro morphologies of the calcareous deposits formed under −1.0 to −1.3 V_SCE_ are shown in Figure 8a–d, respectively. The amount of the deposition increased as the cathodic potential dropped from −1.0 to −1.2 V_SCE_. Meanwhile, the calcareous deposits became loose and porous. When the cathodic potential of −1.3 V_SCE_ was applied to the specimen, many hydrogen bulbs were generated on the specimen’s surface, which influenced the cathodic deposition process. Thus, in Figure 8d, it can be seen that only a few island-like deposits were produced on the specimens. By performing hydrogen permeation of Q460 steel covered with this kind of deposit, it was found that the steady-state current density was smaller than the specimens covered with other deposits, shown in Figure 8g. A smaller steady-state current density means a more minor hydrogen concentration at the subsurface of the specimen. Thus, the deposits formed at −1.1 V_SCE_ can inhibit the hydrogen entry compared with the other deposits. This inhibition of hydrogen entry benefits from the structure of the deposits formed at −1.1 V_SCE_. Based on coupled focused ion beam lithography (FIB) and energy-dispersive X-ray spectroscopy (EDS), it was observed that the calcareous deposits had a calcium-rich inner layer and a magnesium-rich outer layer. Among these, the deposits formed at −1.1 V_SCE_ had a relatively thin inner layer and a condensed outer layer, shown in Figure 8e,f. The validity of this result is proved by some literature. Also, Ou et al. denote that calcareous deposits with pure Mg(OH)_2_ had a better hydrogen resistance compared with the mixture of CaCO_3_ and Mg(OH)_2_ [25]. Xing et al. denoted that the calcium-rich deposits promoted hydrogen adsorption and hydrogen entry, but the magnesium-rich deposits had the opposite effect since the calcium-rich deposits had more pores and interfaces between crystals [26]. In conclusion, deposits formed under −1.1 V_SCE_ benefit from a thin calcium-rich inner layer and a condensed magnesium-rich outer layer, which can inhibit hydrogen entry compared with other deposits in this work. Thus, Q460 steel covered with this deposit had the smallest HE susceptibility.

## 4. Conclusions

The following results are obtained from this work:

1. When performing SSRT in 3.5% NaCl solution and artificial seawater with hydrogen charging, Q460 steel had the smallest HE susceptibility when covered with the calcareous deposits formed under −1.1 V_SCE_. This kind of deposit had a thin calcium-rich inner layer and a condensed magnesium-rich outer layer, which can inhibit hydrogen entry more significantly than the deposits formed under the other three cathodic potentials.
2. A sustained deposition reaction during SSRT in artificial seawater can decrease the HE susceptibility of Q460 steel.

## Figures and Tables

**Table 1:** The composition of the artificial seawater.

Composition	Concentration, mol/L
NaCl	0.42
MgCl_2_	5.46 × 10^−2^
Na_2_SO_4_	2.80 × 10^−2^
CaCl_2_	1.05 × 10^−2^
KCl	9.30 × 10^−3^
NaHCO_3_	2.80 × 10^−2^

**Table 2:** Experimental procedures.

Group	Cathodic Deposition	SSRT Environmental Condition
I	Not proceed	air
II	Proceed	air
III	Proceed	3.5% NaCl, hydrogen charging with 0.5 mA cm^−2^
IV	Proceed	Artificial seawater, hydrogen charging with 0.5 mA cm^−2^

**Table 3:** The mechanic properties of specimens in Group II–IV.

Group	Cathodic Deposition Potential, V_SCE_	YS, MPa	UTS, MPa	δ, %	$I_{\delta }$, %
I	-	470.52	617.97	22.66	-
	-	464.73	615.33	21.94	-
II	−1.0	411.83	614.00	22.95	-
	−1.1	417.94	601.41	23.58	-
	−1.2	367.93	604.36	23.25	-
	−1.3	390.61	610.09	24.68	-
III	−1.0	345.05	634.58	15.36	31.12
	−1.1	393.58	615.17	17.72	20.54
	−1.2	348.61	629.83	15.73	29.46
	−1.3	342.28	628.17	15.96	28.43
IV	−1.0	375.95	625.17	15.68	29.69
	−1.1	388.62	628.67	18.12	18.74
	−1.2	382.68	627.08	16.24	27.17
	−1.3	366.84	635.67	16.76	24.84

## Data Availability

Data are contained within the article.
